# Network-based precision medicine and systems pharmacology

**DOI:** 10.1111/bph.70184

**Published:** 2025-09-01

**Authors:** Arvind K. Pandey, Susan Dina Ghiassian, Joseph Loscalzo

**Affiliations:** 1 Division of Cardiovascular Medicine, Department of Medicine, Brigham and Women’s Hospital and Harvard Medical School, Boston, Massachusetts, USA; 2 Scipher Medicine Corporation, Waltham, Massachusetts, USA

**Keywords:** biomarkers, drug repurposing, network medicine, network pharmacology

## Abstract

The growth in detailed multi-omic profiling has created new opportunities to tailor clinical care and therapy to patient-level variations in disease phenotype. However, efforts towards precision medicine and personalised therapeutics are hampered by limitations in identifying biologically relevant signals that correlate with and underlie disease activity and therapeutic response from these growing arrays of data. These complexities are accentuated further when attempting to translate the new insights in disease pathobiology into new drug targets for treatment. Additionally, understanding how best to reposition existing drugs in the context of new data on disease pathogenesis remains a challenge. Network medicine provides one approach to comprehend these large data sets and identify better the key molecular and phenotypic signals that can function as disease and treatment biomarkers and that can be targeted for therapy. In this review, we discuss basic concepts in the application of network science to biological systems and then build on these concepts to discuss network-based approaches for identifying novel disease biomarkers, elucidating new drug targets and repositioning existing drugs for new indications.

## 1 | INTRODUCTION

The genomic revolution of the late 20th century heralded two paradigm shifts in the study and treatment of human diseases. The first has been the creation of large datasets of human biological and clinical data, driven by the growth of multi-omic technology and computational power. The second has been the pursuit of therapeutic strategies precisely tailored to individualised variation in disease patterns. Both paradigms have proved complementary, with detailed genomic, transcriptomic, proteomic and metabolomic profiling often serving as the basis for understanding variability in disease phenotype among patients and providing a starting point for personalised and precision diagnostics and treatments. This has led to the development of related but distinct fields of personalised and precision medicine. Personalised medicine seeks to achieve a tailored approach to health care based on an individual’s personal profile. For example, a patient’s genomic data can be incorporated to optimise antiplatelet drug selection following percutaneous coronary intervention for myocardial infarction based on predicted drug metabolism in the individual (Magavern et al., 2022). Precision medicine, by contrast, focusses on the development of targeted diagnostic and treatment methods for specific disease contexts or subgroups of deeply phenotyped patients (Ashley, 2016). The growth of targeted treatments for non-small cell lung cancer based on specific cancer genetic changes is representative of such a precision medicine approach (Michelotti et al., 2022). Both precision and personalised medicine have benefited from the growth of ‘omic’ technology, starting with the human genome project and continuing through several subsequent iterations towards a more comprehensive multi-ome.

Even though both paradigms of ‘big data’ and of personalised and precise biomedical care appear to have an almost-harmonised nature, the intellectual framework for understanding and manipulating this synergy is still evolving. Network science has represented one such ‘language’ with which to bridge the gap between the availability of large datasets and the use of these datasets to make novel and meaningful insights into disease pathobiology. The application of network science to the understanding and treatment of disease forms the foundation for network medicine (Barabasi et al., 2011). Network medicine approaches can be applied to better refine pharmacotherapeutic strategies and interventions. The conception of systems pharmacology provided a starting point for thinking about drug effects beyond single-target or single-receptor biology and, instead, assess the effects of a drug across a range of complexities, including cellular, tissue and organismal levels of action. The application of network theory to systems pharmacology has provided new tools for uncovering unique insights on drug targeting and the interaction of drug and disease pathways. Network-informed analyses applied to pharmacology have helped to accelerate new approaches for novel drug target discovery and drug repurposing. Combined with detailed and precise networkbased disease phenotyping through biomarker development, a truly personalised approach to selecting ‘the right drug for the right patient for the right effect’ can be contemplated. In this review, we describe the application of network approaches to biomarker identification, drug target discovery and drug repurposing.

## 2 | BASICS OF NETWORK THEORY AND BIOLOGICAL NETWORKS

### 2.1 | What is a network?

To begin our discussion, a brief overview of networks and their application to biological data is informative. A network refers to a representation of data expressed in the form of *nodes* and *edges* (Figure 1). A collection of nodes and edges defines a network space, and the visual representation of this space is referred to as a *graph*. Nodes can represent any form of information; in biological contexts, this could be a gene, protein, transcript, metabolite, drug, molecule, phenotype, disease or patient. An edge is the connection that exists between two nodes. The nature of this connection can vary depending on the type of network; this can include a physical interaction (such as between two proteins), a target–effector relationship (such as between a drug and its target, or an enzyme and its substrate) or a genotype–phenotype pairing (such as a gene and disease). Whereas edges in some networks simply indicate a connection between two nodes, termed an undirected edge, network representations also can encode additional information in the connection represented by the edge. A directed edge explicitly indicates the direction of information flow from one node to another. An example of this flow would be the connection between a transcription factor and a down-stream regulated protein, indicating the flow of regulation is from the transcription factor to the regulated protein. Another example is a metabolic pathway, where two metabolites are connected using a directed edge to show the action of an enzyme that converts one metabolite into the other. A weighted edge can convey additional information on the quality of the connection between two nodes. For example, in a network space showing ligands and receptors, a weighted edge may indicate binding affinity of a particular ligand to the receptors with which it may interact, with greater weight given to receptors that interact strongly with the ligand versus those that interact only weakly.

Network graphs can take on several different representations (Figure 1f–i). A bipartite graph is a special case where nodes can be divided into two groups, such that edges occur only between nodes of different groups but not the same group. Variations in edge distributions also can occur. A multigraph is a representation in which two nodes can be connected by more than one edge. Alternatively, graphs in which an edge connects a node to itself (pseudograph) or in which an edge can connect more than two nodes (hypergraph) also can be constructed.

### 2.2 | Overview of network topology

For any given network and its representation (graph), mathematical analysis has yielded characteristic features that help to define the properties of the network. These properties, collectively referred to as a network’s topology, can reveal fundamental insights into the nature of networks, including the connectivity of nodes in the network and the overall distribution of edges in a given network space. The number of edges that connect to a particular node is referred to as the *degree* of that node. In the case of networks with directed edges, the concept of the degree can be adapted to refer to edges directed towards a node (indegree) and those directed out of the node towards another node (outdegree). The degree of a node can inform whether the node is a central feature of the network space or is a more peripheral component. Highly connected nodes are referred to as having a high degree and can be termed hubs. The overall distribution of edges per node within a network can provide an organising principle for the large-scale structure of the network. Whereas some have argued that biological networks are characterised by a power-law distribution of degrees, with a small number of nodes with high-degree connectivity (i.e. hubs) and a large number of nodes with low-degree connectivity, this is likely not a universal characteristic, and other types of degree-node distributions have been observed, including a log-normal relationship (Barabasi & Albert, 1999; Broido & Clauset, 2019). This creates structural and functional consequences for the overall network. For example, in scale-free networks, which are characterised by power-law degree distributions, removal of the highly connected nodes is more likely to lead to dissolution of the overall network structure, but because these types of nodes are relatively rare, random removal of any individual node is less likely to affect overall network integrity (Yuan et al., 2016). Several parameters, including network topology, can influence a network’s susceptibility to manipulation; elucidating these points can have important consequences for anticipating controllability of biological systems and is an active area of study (Liu et al., 2011; Lu & Li, 2016; Posfai et al., 2013).

Physical connectivity of nodes to one another can provide one avenue to assess the relative importance and positioning of a node or edge in the overall network. However, it is not the only measure for determining if a node is central to a given network; information flow across a network may alternatively be considered as a distinct measure of connectedness. *Centrality* can provide measures of assessing this type of physical and informational importance of a node or edge to the structure of a network. There are several forms of centrality that can be calculated for a given network (Figure 2). The degree of a node can be referred to as degree centrality. This bases the importance of a node on the degree, or number of connections, a particular node has in the network. Closeness centrality focusses on minimising path lengths between nodes. It identifies the node with the shortest distance to all other nodes in the network. Betweenness centrality identifies central nodes according to the relative share of shortest path lengths that go through a particular node. In effect, it can represent information flow in a network, and it identifies nodes that are highly important in allowing for this flow (connectivity) to occur in the shortest path distance from one part of a network to another. Eigenvector centrality considers the connections of not just a particular node but the connections of the nodes attached to the node. This parameter measures the ‘connection of connections’ a particular node may have, with higher measures of centrality assigned to nodes with neighbours that are themselves highly connected.

### 2.3 | Network modules

Utilising these concepts, areas within networks can be identified in which nodes may relate more closely to each other within a particular ‘local’ group, either physically or functionally, than to other nodes in the network. These subnetworks, or *modules*, represent a key component that allows translating the mathematical concepts of network science into a biologically relevant context (Figure 1j). Biological systems can mirror such a modular nature, providing a strong correlate to understanding these graph features (Hartwell et al., 1999). It should be noted that network modules can in some cases be identified as topologically distinct regions within the network graph; however, this is not required. Topological clusters can be one subset of the broader group of network modules but they are not the only type; a module also can share nodes that cross several physical and/or functional domains of a network. A *disease module* is a specific type of subnetwork comprised of all nodes necessary for the manifestation of a disease (Menche et al., 2015). Disease modules can provide a more comprehensive framework from which to understand how relationships between subnetworks in a network space can correlate to biological systems. Disease modules for two separate diseases that are in close network proximity may be affected by a perturbation to a similar extent because the network space they occupy is similar. This is analogous to how closely related disease processes may share common alleviating and exacerbating factors because of shared pathobiological features (Menche et al., 2015). Distinguishing disease modules from other network motifs and subnetworks is therefore key to be able to apply network medicine approaches to understanding new diagnostic and therapeutic approaches. The general approach for this methodology is to identify disease genes or proteins and then elucidate how these disease nodes assemble into the larger structure of a disease module. These methods encompass a wide range of approaches, including linkage analysis to identify direct interaction partners, seed connector algorithms which start with a known set of disease-associated genes or proteins (seeds) and then computationally identifies other disease genes or proteins comprising the disease module, random walker algorithms which rely on ease of ‘diffusion’ of information through a network, approaches evaluating the quality of connections between disease nodes rather than quantity to identify functionally related disease subnetworks across a network’s topological space, and communality analysis using the Leiden algorithm which attempts to optimise partitioning and community detection in a network space (Bersanelli et al., 2016; Ghiassian et al., 2015; Kohler et al., 2008; Pandey & Loscalzo, 2023; Sebastian et al., 2023). The resulting identification of disease-related nodes and disease modules is a foundational step to be able to understand and track better the status of a disease and its response to treatment through network approaches. One key application is for the identification of disease biomarkers.

## 3 | NETWORKED-BASED IDENTIFICATION OF BIOMARKERS

Biomarkers are integral to modern medical care, serving as indicators of normal biological processes, disease activity, risk for future pathologic processes and/or therapeutic response. Whilst straightforward in concept, the complexity of biological systems represents a challenge in the identification of true surrogates for these endpoints. A single, linear mechanistic pathway that links disease, biomarker and outcome may not exist; rather, biomarkers may only partially correlate with disease activity or treatment effect (Figure 3) (Califf, 2018; Fleming & DeMets, 1996). This complexity has increased exponentially with the growing availability of multi-omic data because the relationships between individual data points with larger phenotypic or therapeutic trends may not be evident immediately by the identification of an individual data point itself. Network approaches offer a potential strategy for addressing this growing complexity in the development of new biomarkers. The elucidation of specific network features that correlate with disease representations (disease modules in the protein–protein interactome, for example) lends itself to the idea that there exists a subset of network components that encode key pieces of information that dictate the nature or relationship of the larger disease network. Translating these key network components from a network space to a biological context would serve to identify biomarkers that could theoretically provide highly precise diagnostic, phenotypic, or therapeutic information for tracking disease status and treatment response.

### 3.1 | Network topology in biomarker discovery

The topological characteristics of nodes within a biological network can be one basis by which potential biomarkers can be identified using network analysis. Measures of centrality are commonly used to harness this topological information. One criterion can be degree centrality, which identifies hubs in a network as nodes with high degrees and, thus, comparatively greater connections to other nodes. One study utilised a degree centrality-based approach to identify candidate microRNAs (miRNA) in patients with glioma who underwent miRNA and mRNA profiling (Li et al., 2013). After constructing a bipartite miRNA-target mRNA regulatory network with directed edges indicating the direction of regulation, the authors identified three miRNA signatures that correlated strongly with outcomes and specifically noted that hub proteins drove the risk stratification. Among the hubs present in all three signatures, they identified two miRNAs that on subsequent functional validation showed broad regulatory activity over several biological pathways relevant for cancer progression. This approach of looking at network hubs as biomarkers has been applied to the role of non-coding and competing endogenous RNAs more broadly (Paci et al., 2014; Paci & Fiscon, 2022) as well as other cancers (including lung cancer [Xu et al., 2020], colon cancer [Nishihara et al., 2017] and cervical cancer [Reza et al., 2022]) and cancer risk prediction (Kasera et al., 2023). Other measures of centrality can alternatively be used as topological filtering mechanisms to identify nodes that can serve as putative biomarkers. Ozugur and colleagues (Ozgur et al., 2008) assessed how well different measures of centrality performed in identifying genes related to prostate cancer. They found that eigenvector centrality and degree centrality perform well in identifying known genes that associate strongly with prostate cancer, whereas betweenness centrality can better identify previously unknown genes that may merit further investigation. Another study utilised three indices of centrality (degree, betweenness and closeness) to identify four amino acids (lysine, cystine, glycine, and tryptophan) as hub metabolites that are decreased in the plasma of young men with hypertension (Wang et al., 2015). Centrality assessment also has been applied to identify potential early-stage biomarkers among miRNAs for cardiovascular diseases (Ahmed et al., 2022), and betweenness centrality was used to identify the protein NEDD9 (neural precursor cell expressed, developmentally down-regulated 9), as an important circulating biomarker of endothelial fibrosis in pulmonary arterial hypertension (Samokhin et al., 2018; Samokhin et al., 2020). Other node prioritisation methodologies beside centrality can be used to identify putative biomarkers. For example, one study utilised guilt by association (GBA) to prioritise genes across several different cancer subtype-specific networks (including breast, lung and kidney cancers) constructed using the data from The Cancer Genome Atlas and the Genotype-Tissue Expression project (Zhang et al., 2023). GBA prioritises nodes (genes) based on relationships linking a particular gene with known disease-causing genes; nodes that have strong connections with known diseasecausing genes are more likely to be involved in the disease process themselves. The identified genes in the study demonstrated a strong ability to both discriminate cancer cases from normal controls and to predict survival time in cancer patients.

### 3.2 | Modular network biomarkers

Another approach to biomarker identification focusses on the collective expression of several nodes that form a subnetwork or module. This type of ‘network biomarker’ is typically constructed by combining gene expression data with the protein–protein interactome to identify collections of nodes that correlate with diseases. The need for this type of approach can be particularly notable in heterogeneous disease states like cancer, which typically have several possible drivers as a function of genomic context that can vary from one study to another (Fisher et al., 2013; Loeb et al., 2003). The constructed networks are, thus, likely to differ from one study to another, making the identification of critical single-node biomarkers unlikely from such a heterogeneous background. Development of modular network biomarkers takes this heterogeneity as a starting point and, rather than look at individual nodes (genes or proteins) within networks, network modules are identified that represent functional pathways or other meaningful clustering within the larger network. The functional pathways and relationships that underlie this clustering are more likely to remain consistent across datasets and, accordingly, modules based on these pathways have greater reproducibility and better correlate with disease presence. This methodology utilising functional subnetworks applied to breast cancer (Chuang et al., 2007) demonstrated greater concordance of functional pathways between two separate datasets compared to the degree of concordance observed when examining the gene lists of the two datasets in isolation. Moreover, the identified subnetworks correlated well with typical characteristics of breast cancer and better identified tumour expression profile as representing metastatic or non-metastatic tumours, identifying these subnetworks as putative ‘network biomarkers’ for both disease presence and activity. This approach has been extended to several other cancers, including lung cancer, colon cancer, ovarian cancer, uterine cancer and glioma (Al-Harazi et al., 2021; Hofree et al., 2013; Wang & Chen, 2011; Zhang et al., 2020), and it has been refined to better identify cases and controls in the setting of breast cancer (Xin et al., 2015). Additionally, Jin and colleagues have demonstrated that a strategy incorporating multiple network biomarkers performed better than those with single or paired network biomarkers for cardiovascular diseases (Jin et al., 2008).

Beyond a simple binary designation of diseased or non-diseased state, biological systems can be characterised by a gradient from a healthy to a disease state over time. Within this temporal transition, critical time points, called tipping points, herald the transition from a reversible to an irreversible progression towards the disease state (Shi et al., 2021). A biomarker for this tipping point state, a so-called ‘early warning signal’, could predict which patients may benefit most from targeted intervention to alter the disease trajectory away from this irreversible decline towards disease. This goal has led to the growing interest in dynamic network biomarkers, which attempt to identify these tipping points using networks constructed from omic and multi-omic data analysed over time. Chen and colleagues described a methodology using pairwise Pearson correlation coefficients to identify a dominant group of nodes within a network that showed a marked increase in correlation among themselves and a decrease in correlation with other nodes outside of the dominant group as a system reaches the tipping point (Aihara et al., 2022; Chen et al., 2012; Liu et al., 2012). This dynamic network biomarker approach has been applied to models of lung injury (Chen et al., 2012), the development of hepatitis B virus-induced hepatocellular carcinoma (Chen et al., 2012), hepatocellular carcinoma metastasis (Yang et al., 2018), differentiation of embryonic stem cells to other cellular fates (Li et al., 2023), as well as other human cancer and metabolic phenotypes (Han et al., 2022; Li et al., 2014; Liu et al., 2013). Dynamic network biomarkers illustrate the importance of incorporating time in understanding the transition from a non-diseased network to a diseased network space. These types of biomarkers also highlight the idea that is evident in clinical practice that disease natural history is an important consideration for understanding prognosis and therapeutic responsiveness. More comprehensive inclusion of these temporal factors will enhance the predictive power of network medicine approaches in general for understanding disease biomarkers and for its application to disease therapeutics.

## 4 | NETWORK PHARMACOLOGY AND PRECISION THERAPEUTICS

The use of network analysis to develop more precise methods for disease phenotyping and biomarker identification naturally leads to the question whether these approaches could be used to refine drug development and targeting. Precision phenotyping can create opportunities for precision therapeutics: a better understanding of the genes, proteins, metabolites and other biological features that define a disease state can create new opportunities for targeting those features for treatment. A precision-based approach and mindset to pharmacology and drug development also has the potential to improve understanding of on- and off-target effects of existing drugs. This opens new possibilities for repurposing and repositioning drugs for novel indications and for better adjusting drug regimens to avoid side effects.

Fundamental to this precision approach to drug therapy is a more comprehensive understanding of a drug’s pharmacology that goes beyond the target against which it was designed or even the molecular pathway it affects. A 20th century Ehrlichian approach to drug development, attempting to find a single highly specific and highly specialised ‘magic bullet’ to cure a disease (the ‘one disease-one target-one drug’ paradigm), has led to increasing stagnation in drug development over the last several decades. When normalised to inflation-adjusted yearly research expenditures, drug development efficiency has declined nearly 80-fold from 1950 to 2010 (Scannell et al., 2012). This decline has been termed Eroom’s Law, to contrast it with Moore’s Law in computing which describes the exponential growth in the number of transistors in an integrated circuit over time. The causes driving Eroom’s Law are multifactorial and complex, encompassing issues such as regulatory risk tolerance, financing of drug research and development, and relative efficacy of new versus existing drugs. From a basic research perspective, drug target identification and druggability of identified targets are important considerations. Although we will focus on drug target identification in this review, the issues around druggability are multi-layered and include biophysical and biochemical considerations of putative targets. The share of proteins encoded by the genome that are considered druggable is estimated as low as 15% (Rodgers et al., 2018), indicating a large gap that can impact the process of going from target identification to drug development. Among the challenges in the drug development process, there is increasing difficulty in identifying suitable targets for therapy that are effective whilst also having a minimal side effect profile. Investigational drugs have high attrition rates during clinical drug development; estimates suggest as many as 90% of all drug candidates do not reach market due to their failure to satisfy the basic tenets of efficacy and safety, with the most frequent cause being unacceptable drug toxicology (Hughes et al., 2011; Sun et al., 2022; Waring et al., 2015). This level of failure would appear to be paradoxical at first glance given the increased specificity with which drug targets are initially identified, often utilising increasingly specific and targeted studies in specific model systems that are able to functionally isolate a particular mechanism or mechanistic pathway. However, this increased ability to selectively isolate and manipulate biological processes has likely hampered a more holistic understanding of how such a target or pathway may integrate within a biological system at the organism level.

In a more ecumenical approach to understanding the pharmacology of a drug, efficacy and toxicity are not believed to be the result of single, linear drug–target interactions but, rather, represent emergent properties arising from a network of interactions involving the drug, the biological system and the broader environmental context (Huang, 2002). An emergent property is one that cannot be predicted solely based on the components of the system; instead, it represents a higher level, integrated response that ‘emerges’ from the function and interaction of several or all individual components. The addition of greater biological information utilising such a network approach could help overcome both the efficacy and toxicity issues by providing a new tool for identifying targets for drug development more effectively while avoiding side effect-causing targets. The rationale for such an approach is illustrated by studies in yeast demonstrating how addition of greater biological context can impact the potential suitability of a gene or protein as a drug target. Traditional knockout studies initially identified that only approximately a third of the yeast genome was necessary for survival and/or growth fitness of the organism (Deutschbauer et al., 2005; Giaever et al., 2002; Winzeler et al., 1999). This would suggest that this minority portion of the yeast genome would be the most relevant for considering potential therapeutic interventions because this subset would have the most biologic effect. However, when the yeast’s environment was perturbed, or modified, from the idealised conditions of typical high-nutrient laboratory growth, another 60% of the genome demonstrated a meaningful impact on the organism’s growth (Hillenmeyer et al., 2008). This increased complexity, not evident from initial evaluation of gene and protein function, is an ideal target for network-based approaches for understanding drug targetability and therapeutic effect. Such a network pharmacology approach attempts to understand all possible targets of a drug, not just the molecule or pathway it was designed to affect, having the ability to incorporate population and patient level genomic, epigenomic, proteomic and environmental data such that the complexity of the overall system can be better understood and harnessed for improving both drug efficacy (Mellors et al., 2020; Shanthamallu et al., 2024) and avoiding drug toxicity (Paci et al., 2022).

## 5 | DRUG TARGET IDENTIFICATION USING NETWORK APPROACHES

Network approaches can identify potential biomolecular targets that could be predicted to have better efficacy in manipulating a pathophenotype beyond what may be predicted from simple protein binding or linear pathway analysis of signal transduction, receptor-ligand interactions and metabolic pathways. Network-based methods that incorporate disease module information for target selection can form the basis for identifying precision therapeutics that not only target the most critical biological components of a disease but that also minimise side effects by avoiding actions on non-disease-module nodes.

### 5.1 | Network nodes as drug targets

Network-informed drug target identification can be tailored based on the pathobiologic scenario that is being targeted to better elicit the desired therapeutic response (Figure 4). Such an approach acknowledges that different diseases have distinct pathophenotypes and, by extension, distinct disease module characteristics in a network space. Using this conceptual approach, a ‘central hit strategy’ can be taken for diseases with cells that are rapidly dividing or that are highly plastic (Csermely et al., 2013). Drug targets in this case would be chosen from nodes that have high centrality features and that would be predicted to have large effects on disease module and network integrity. Clinical scenarios where this approach can be applied include infectious diseases and cancer (Barve et al., 2012; Folger et al., 2011; Singh et al., 2007). For example, Singh and colleagues used this type of strategy to identify key metabolic pathways in *Entamoeba histolytica*, one of the leading causes of parasitic deaths in the world, that could be targets for therapy, including peptidoglycan and streptomycin biosynthesis (Singh et al., 2007). Biological networks for these states share several features with highly dynamic systems, which are characterised by high degrees of robustness and resiliency to perturbation (Kitano, 2007). A robust network is one in which measurable components are resistant to change despite changes in intra-network organisation or topology. These features are positively correlated with the concept of network entropy, which conveys the level of randomness in the information encoded in the network, providing a measure of network complexity (Demetrius & Manke, 2005). Nodes in the network that more disproportionately contribute to the overall network entropy are more likely to have large biological effect because they are more likely to affect network and, hence, biological resiliency (Manke et al., 2005). Choosing these more entropic nodes as drug targets in highly dynamic networks, as in the case of cancer or infectious diseases, would be predicted to better overcome network inertia and impact or disrupt a disease module, and thereby counteract the network’s (and, by extension, the biological system’s) resiliency in order to elicit a therapeutic effect (Guo et al., 2021; Prado-Prado et al., 2011; Teschendorff & Severini, 2010; West et al., 2012).

A second approach for selecting drug targets is to target nodes that modify or influence central nodes without directly aiming for the high centrality nodes themselves. The finding that currently available drugs generally impact only a subset of a disease module with limited spill-over to adjacent network components and do not disrupt the entire module provide support for the applicability of such a strategy (Guney et al., 2016). This ‘network influence strategy,’ as termed by Csermely and colleagues (Csermely et al., 2013), is likely better suited for pathologic conditions affecting differentiated cells where disease conditions are more likely to arise from specific maladaptive functions of these cells. In this case, disruption of the cell’s global state, as may happen by targeting a highly central node, may negatively impact cell viability and lead to enhanced cytotoxicity. This may be undesirable given that highly differentiated cells may not be easily replaced and, thus, such a large effect would be detrimental rather than beneficial in a disease state. Conceptually, this type of network can be considered as ‘rigid’ and contrasts with more ‘flexible’ networks of cancer and infectious diseases that would be targeted by a central hit strategy. Whereas the concept of rigidity is best understood intuitively for physical objects, functional rigidity or flexibility can be considered in the context of biological networks (Gaspar & Csermely, 2012), in particular dynamic networks, to indicate the number of states that a system can reach in response to external forces or factors. In this framework, a rigid network has fewer possible configurations to take on in response to a given perturbation. Thus, a drug impacting a central node in a rigid network may cause an effect that propagates quickly and broadly throughout the network space, correlating with significant side effects and potentially lethal toxicities in the biological system (Csermely et al., 2013; Perez-Lopez et al., 2015). Targeting neighbouring nodes instead could allow for smaller effects that can more effectively modulate the biological impact of the drug while dispersing side effects. As an example of this type of strategy, Voitalov and colleagues used three types of network modules in ulcerative colitis (modules containing disease genes, genes which are altered in treatment responders, and genes which are targeted by current therapies) to identify new targets which influence the disease phenotype in this condition (Voitalov et al., 2022). A network influence approach is analogous to the concept of how adult disease genes, and embryologically lethal disease genes, tend to be distributed in a human disease network space. Embryologically lethal disease genes are generally found in hub positions in biological networks, and, thus, mutations in these genes exert wide-ranging effects on the integrity of the network space and consequently the biological system; in contrast, adult disease genes tend to be in the periphery of the network and have relatively smaller and more targeted effects (Pandey & Loscalzo, 2023).

A related method to achieve a similar result as the network influence strategy is to use a multi-drug approach. In this case, each drug of a multi-drug regimen is targeted to a particular pathobiological component (i.e. network feature), creating smaller partial effects that in summation across all the drugs in the regimen produce the desired therapeutic effect. Network approaches can help to delineate out how a multi-hit strategy can impact network robustness and disease pathobiology to mediate the desired effect on a disease process, particularly those with a paucity of current effective therapeutics like stroke (Ágoston et al., 2005; Araujo et al., 2007; Casas et al., 2019; Luni et al., 2010; Wang et al., 2012). Theoretically, this approach could reduce side effects by allowing for lower concentrations of individual drugs and better targeting of multiple less-central nodes. Multi-drug regimens already have clinical acceptance in several disease conditions, such as guideline-directed medical therapy for heart failure and highly active antiretroviral therapy for human immunodeficiency virus infection. Network approaches that can similarly identify other scenarios and may benefit from a multi-drug approach have the potential to improve treatment efficacy whilst also providing greater insights into potential side effects and relative effectiveness of individual drugs, a goal that is challenging in current multi-drug treatment approaches (Malgie et al., 2023).

### 5.2 | Edgetic drug targets

Another candidate for network-informed drug target selection is to identify edges that could provide therapeutic effect, rather than only focussing on nodes. In this approach, a putative drug would selectively target a subset of edges of one or more nodes to mediate its effect, rather than globally affecting the node and all its connections (edges). Such an edgetic drug would spare non-disease related edges to limit side effects. The nature of an edge is dependent on the construction of the biologic network, and, thus, edgetic drug targets can take on several different forms. In the context of protein–protein interaction networks, edgetic drug targets affect a subset of protein interactions of a particular node (protein). Whereas development of such protein interaction modifiers has been slow, there is great potential for this type of targeting strategy because the human protein–protein interactome is predicted to contain at least 650,000 interactions (Stumpf et al., 2008). Additionally, most mutations in Mendelian diseases disrupt protein–protein interactions, with a majority of these disruptions occurring in an edge-specific manner rather than modulating all interactions of the affected gene product (Sahni et al., 2015; Zhong et al., 2009). Determining a genetic mutation’s impact on a protein product’s ‘edgotype’ can provide clues as to how to target edgetic drugs to specifically affect certain interactions of proteins, promoting beneficial protein–protein links whilst impairing pathogenic interactions. One study found that edgetic mutations are more likely to cluster around surfaces exposed to the environment in the three-dimensional structure of the product protein, although mutations that affect all interactions of a protein (and are not edgetic) tend to affect overall molecular hydrophobicity, which is more likely to be globally destabilising for the protein (Sahni et al., 2015). Similar to the theme observed with the central hit strategy, certain edges can have greater effects on network robustness and resiliency, and certain nodes have more edgetic effects than others; these subsets of nodes and edges serve as attractive edgetic drug targets for diseases with increased network resiliency, such as cancer (Kataka et al., 2020; Truong & Kwon, 2017). Likewise, edgetic drugs can exert network influence-type effects given the targeted nature by which they affect a node’s interaction with other nodes. These varying influences are important considerations when considering how to adapt pre-clinical data on drug targets, which may be based on gene silencing or animal knockout studies, for pharmacologic modulation, which may exert a more nuanced effect. Anticipating whether a drug will elicit a nodal effect (and whether this effect will follow a central hit or network influence paradigm) or an edgetic response can have significant implications for expected therapeutic efficacy and side effect potential.

Several therapies can be considered edgetic in their pharmacodynamic effects. tirofiban functions as a small molecule inhibitor disrupting the specific interaction of fibrinogen with glycoprotein IIb/IIIa to impair platelet aggregation (Hartman et al., 1992). Tafamidis acts as a stabiliser of the native transthyretin tetramer, minimising pathologic protein–protein interactions from partially denatured transthyretin monomers in patients with predisposing mutations (Said et al., 2012). Other drugs harnessing protein–protein interactions for therapeutic effect are under development. These include nutlin inhibitors, which work to activate p53 tumour suppressor activity in cancer by impairing its physical interaction with the p53 inhibitor and E3 ubiquitin ligase MDM2 (Shen & Maki, 2011), and drugs targeting bromodomains, which mediate protein interactions through interactions with acetyl-lysine groups (Filippakopoulos & Knapp, 2014).

## 6 | NETWORK APPROACHES FOR DRUG REPURPOSING

Given the declining efficiencies and prolonged timelines associated with the drug development process, repurposing existing drugs has been an area of focus and research to expand the repertoire of potential therapeutics using drugs that have already demonstrated reasonable toxicity and safety profiles in humans and that have manufacturing processes already in place. Whereas drug repositioning can still be associated with unpredicted side effects and efficacy issues because of drugs being used in new pathobiological contexts and/or new dosages, drug repositioning efforts have the potential to decrease a typical drug development life cycle by years whilst also providing a potentially cost-effective method for finding therapies for rare diseases (Ashburn & Thor, 2004). Historically, drug repositioning was more likely to occur because of accidental or serendipitous discoveries of alternative uses; the case of minoxidil, initially studied as an antihypertensive but subsequently found to stimulate hair growth, is one illustrative example. However, systematic approaches are now being applied, and drug repositioning can be performed based on several strategies. Broadly, these can be grouped into identifying new uses based on similarities in disease pathobiology, drug targets or drug action (Figure 5) (Parisi et al., 2020).

### 6.1 | Conceptual frameworks for drug repurposing

The fundamental premise of repurposing efforts is that there are shared disease pathways, motifs, and/or mechanistic underpinnings that allow more than one chemical to elicit a desired biological response, or that allow one chemical to mediate several desired biological responses. Disease-based repositioning involves repurposing an existing drug for one disease to be used for a second, closely related, disease condition. Examples of this strategy include the repurposing of nifedipine, which is utilised primarily as an antihypertensive, for the treatment of vasospasm in Raynaud’s disease, or expanding the use of the checkpoint inhibitor ipilimumab from melanoma, for which it was originally approved, to other cancers. Although repositioning based on disease pathobiology tends to be the most common strategy (Parisi et al., 2020), this approach is not foolproof and can highlight important pathobiologic differences between what may seem to be related pathophenotypes when repurposing does not work as hypothesised. For example, although β-adrenoceptor blockers have shown remarkable efficacy in the treatment of heart failure with reduced ventricular ejection, they have not produced similar benefits on disease outcomes in heart failure with preserved ejection fraction, providing a window into the differing mechanisms underlying these two forms of heart failure.

Repositioning based on targets identifies a known drug target that plays an important role in other diseases. The drug for that target can be repurposed to treat the spectrum of other diseases sharing that common link. An example of this target-based repositioning is sildenafil, an inhibitor of phosphodiesterase 5 (PDE5), that was initially developed for the treatment of angina pectoris by enhancing nitric oxide (NO) bioavailability to promote vasodilation. Sildenafil was subsequently repurposed for other diseases that are characterised by impaired NO signalling, including erectile dysfunction and pulmonary hypertension.

Repositioning based on drug action involves identifying new molecular targets for a drug that can be utilised in the treatment of other diseases. One example of this approach is sotalol; as a non-selective β-adrenoceptor blocker, it can reduce the contractility of cardiac myocytes like other beta blocker medications. However, sotalol also was found to inhibit potassium ion channels and enhance cardiomyocyte repolarisation, allowing it to function as potent anti-arrhythmic medication, as well. Although drug-based repositioning has been slower to develop, there is significant potential. One study constructed a map of all chemicals known to have human biological targets, and of the >275,000 chemicals identified, 35% of the compounds were found to have more than one biological target (Paolini et al., 2006). Additionally, when these authors created a ‘poly-pharmacology network’ in which nodes (proteins) shared an edge if they had a common interacting compound, the 700 proteins that were mapped demonstrated clustering based on similarity in structure and function, indicating that structurally similar proteins shared similar interacting chemical compound ligands. Thus, a particular drug with a known protein target may also be a candidate for repurposing for diseases involving the protein family to which the original target belongs. This type of large-scale drug-based repositioning approach has been experimentally verified using in vitro and in vivo models, although there is inherent attrition in candidate drugs going from a set of computationally proposed drug–target interactions to an experimentally verified one (Keiser et al., 2009).

Among these conceptional frameworks, drug–target and drug–disease based repositioning have seen the widest adaptation to network-based approaches. We discuss these approaches for drug–target and drug–disease based repositioning strategies in the subsequent sections.

### 6.2 | Drug–target based repurposing

Network approaches for drug repurposing help to integrate large amounts of data pertaining to disease phenotype, drug target, and pharmacologic information to identify and prioritise drugs that can be candidates for repurposing. One approach starts with constructing a bipartite drug–target interaction network, in which drugs and targets form the two groups of nodes, and an edge represents a validated interaction between a drug and a target (Yildirim et al., 2007). With this starting point, new edges can be predicted in this network space, which would identify novel drug–target interactions. Methodologies including machine-learning approaches based on probabilistic soft logic (Fakhraei et al., 2014) and probabilistic matrix factorisation (Cobanoglu et al., 2013) as well as supervised inference (Cheng et al., 2012) have been applied to bipartite drug–target interaction graphs to predict novel edges. Another study designed an algorithm, termed SITAR, to predict drug–target interactions based on similarity measures between drugs and drug targets (Perlman et al., 2011). Relative similarities between drugs (drug–drug similarity) were first determined and scored based on five distinct attributes: these included drug side effects, drug response gene expression profiles and pharmacodynamic-based drug ontology using the Anatomic Therapeutic Chemical classification system. Target gene similarities were scored using three features (genetic sequence, network distance of gene products when mapped to a protein–protein interactome and Gene Ontology terms). The drug–drug and gene–gene similarities were then combined to determine rules that identified true versus false drug–gene associations using the similarity scores, and then using these rules, the authors predicted novel drug–gene associations.

### 6.3 | Drug–disease based repurposing

Disease-based network repositioning methods often involve a drug–disease network; this is analogous to a drug–target network in that it is a bipartite graph, but instead of drug targets, the second set of nodes are diseases, and edges represent a verified relationship between a drug and a disease. Chiang and Butte used a method based on GBA to identify new drug repositioning candidates for new disease indications (Chiang & Butte, 2009). Using the GBA principle, nodes that are highly connected are more likely to be functionally similar. Adapted to drug–disease relationships, if two diseases are highly connected, they are more likely to share therapeutic drugs. The authors used this concept to identify novel repurposing predictions for known drugs, such as repurposing atorvastatin for use in a number of cancers. Chen and colleagues used network topology-based inference methods applied to a drug–disease network space to identify new diseases to be targeted by existing drugs (Chen et al., 2015). Another methodology by Paci and colleagues, called SAveRUNNER (Fiscon et al., 2021; Fiscon & Paci, 2021), used topological characteristics to identify novel drug–disease relationships based on the premise that drugs that affect a particular disease will likely be close to the disease module of that disease in network space. The input for this algorithm is a drug–target network and a disease-gene network; the algorithm then calculates the drugs that are in the vicinity of a disease based on a measure of network similarity to output a drug–disease network with novel drug treatment predictions. The authors subsequently refined this approach to incorporate side effect information by having the algorithm maximise the distance between drug repurposing candidates and side-effect genes (Paci et al., 2022). The investigators who developed the SITAR algorithm to predict new drug–target interactions designed another algorithm that they termed PREDICT for drug repurposing using disease-based repositioning (Gottlieb et al., 2011). The authors took a similar approach as for the SITAR algorithm and determined drug–drug similarity and disease–disease similarity scores, combined these rules to identify known drug–disease associations, and then used these rules to predict novel drug–disease associations. Then, they verified how closely these predictions aligned with real-world attempts at expanding drug use indications by comparing how many of their predicted drug–disease associations were being utilised in phase I–IV clinical trials.

Another group of methodologies rely on drug-induced gene expression profiles. The most extensively utilised resource for this is the Connectivity Map (CMap), a compendium of differential gene expression profiles in response to drug-and genetic-based perturbations across multiple cell lines (Lamb et al., 2006; Subramanian et al., 2017), although other similar datasets have been compiled and utilised to study drug repositioning (Hu & Agarwal, 2009). Using CMap, a gene expression profile for a given disease or drug-treated state can be experimentally determined and then compared to the collated drug-treated gene expression signatures in CMap to identify drug treatments that are most similar to, or different from, the given experimental profile. Taking the case of an experimentally determined disease state expression profile, an anti-correlated drug profile in CMap may be considered as a potential therapeutic that could be used to potentially ‘push’ the genetic profile of the disease back towards a normal state, and, thus, can be a candidate for drug repurposing to treat the disease (Iorio et al., 2010; Iorio et al., 2013). This methodology has been applied to predict new therapies, including the use of topiramate for inflammatory bowel disease (Dudley et al., 2011) and cimetidine for lung adenocarcinoma (Sirota et al., 2011). However, for both of these repurposing predictions, follow-up studies have been mixed regarding the robustness of the therapeutic responses for the drugs (Crockett et al., 2014; Kandela et al., 2017; Silva et al., 2022). This suggests important caveats when considering this type of approach. For example, it is possible that the genetic and transcriptomic profiles are not causative but are merely correlative, such that simply matching expression profiles will be insufficient to mediate a therapeutic effect. Additionally, the expression patterns in cell lines may not adequately match human-derived pathological samples, resulting in possible false positives and negatives. Experimental and clinical validation studies are critical to assess the accuracy of the predicted outcomes so that prediction models and bioinformatic approaches can be further refined and enhanced.

### 6.4 | Heterogeneous network-based drug repurposing

Network approaches that combine drugs, targets and diseases have been undertaken to construct a more comprehensive network space from which to make predictions on drug repositioning. Yamanishi and colleagues incorporated information on drug structure and target protein amino acid sequence (constructing a combined ‘pharmacological space’) along with network topological analysis of the drug–target network graph to make new drug–target predictions (Yamanishi et al., 2008). Utilising a supervised learning approach to identify nearest neighbours based on network topology, they made several predictions for candidate drug–target interactions that incorporated structural information of both the drugs and their protein targets, and they identified some predictions that have previously been experimentally verified. Berenstein et al (2016) also utilised drug structural information via a multilayer network incorporating drug–drug chemical similarity, protein drug targets and protein functional affiliations. These authors identified novel drug repurposing predictions based on the structural and functional similarities of the targets. Luo and colleagues incorporated multiple pieces of information, including drug–drug, drug–disease, drug–side effect and drug similarity networks to create a combined heterogenous network from which to predict novel drug–target interactions and drug repurposing candidates (Luo et al., 2017). Another approach evaluated all possible connections going from a drug to a disease through causal chains of drug–target, target–pathway, pathway–downstream gene, gene–disease, and drug–disease relationships (Li & Lu, 2013). Using causal inference, the authors identified drug repurposing candidates using this multilayered network. Disease module identification also can aid in drug repurposing. Combining drugs, drug targets, and disease genes for a particular pathophenotype can help to identify drugs that may be repurposed for the disease process based on the idea that a drug may be repurposed if it is close to that pathophenotype’s disease module (Wang & Loscalzo, 2016). The NedRex platform expands on this idea by combining several data types and sources to create a heterogeneous network covering drugs, drug targets, genes and disease relationships (Sadegh et al., 2021); this platform can be used to reconstruct disease modules using seed genes or gene expression data and then identify drug repurposing candidates. The applicability of heterogenous multi-modal networks for drug repurposing was recently demonstrated with the use of these approaches for identifying novel repositioned drug candidates for the treatment of COVID-19 (Stolfi et al., 2020; Wang et al., 2021).

## 7 | CONCLUSIONS AND FUTURE DIRECTIONS

The application of network and graph theory to biomedical science has created new opportunities to understand the growing multi-omic data sets that are defining modern approaches to scientific inquiry. Ongoing efforts towards precision and personalised medicine necessitate a more detailed view of disease and pharmacologic therapies to understand clinically meaningful subgroup and individual variation within larger disease entities. Whereas ‘big data’ have provided one source for defining these more granular pictures of pathobiology and pharmacodynamics, harnessing and, more importantly, analysing this growing volume of data in a clinically meaningful has remained a challenge. Network medicine provides a new and powerful strategy by which to make these large datasets more tractable to yield actionable biological and therapeutic information, spanning the entire spectrum of disease definition, markers of disease activity and targets for disease treatment (Table S1). In the future, these network approaches could inform precision disease profiling to identify personalised therapeutic strategies tailored to an individual. Using a patient’s own biological data, an individual’s network space could be analysed to identify the critical proteins (nodes) or groups of proteins (modules) that are responsible for that patient’s specific disease phenotype or propensity for that disease phenotype. Network pharmacology could then be applied to identify the therapies that would most likely provide effective therapeutic benefit whilst minimising side effects. Network analysis also provides hope for improving drug repurposing efforts. The recent COVID-19 pandemic has provided a case study for these approaches, both the opportunities and challenges (Morselli Gysi et al., 2021; Wang et al., 2021; Wang et al., 2023; Wang & Loscalzo, 2023; Zhou et al., 2020), but one indisputable observation is the speed with which these computational tools can be deployed. The rapidly growing adoption and incorporation of artificial intelligence (AI) methods into scientific discovery will further enhance these network-based approaches with new methodologies that further improve the accuracy and efficiency. Machine learning and deep learning methodologies are already being applied to identify network-based biomarkers, identify new drug targets, repurpose existing drugs for both prevalent and rare diseases, and predict responses to therapy, and identify side effects and toxicities of drug treatments (Das, 2024; Gangwal & Lavecchia, 2025; Huang et al., 2024; Kamya et al., 2024; Kong et al., 2022; Lacalamita et al., 2023; Rodriguez et al., 2021). Moreover, the incorporation of explainability algorithms (Yuan et al., 2023) into workflows, which seek to make the reasoning behind the output of AI methods interpretable and less of a ‘black box’ as is the case in traditional AI algorithms, may allow for greater understanding of mechanistic underpinnings of AI-based predictions to facilitate bench-side validation studies and result in novel disease and drug insights (Jiménez-Luna et al., 2020; Wong et al., 2024). While network methodologies continue to evolve towards ever more meaningful biological predictions, the ongoing expansion of multi-omic profiling provides a strong rationale for the continued growth of these network-based diagnostic and therapeutic strategies.

### 7.1 | Nomenclature of Targets and Ligands

Key protein targets and ligands in this article are hyperlinked to corresponding entries in http://www.guidetopharmacology.org , and are permanently archived in the Concise Guide to PHARMACOLOGY 2021/2022 (Alexander, Fabbro, Kelly, Mathie, Peters, Veale, Armstrong, Faccenda, Harding, Pawson, Alexander, et al., 2021; Alexander, Fabbro, Kelly, Mathie, Peters, Veale, Armstrong, Faccenda, Harding, Pawson, & Southan, 2021).

## Supplementary Material

Pandey___Loscalzo_2026_Supplement

Additional supporting information can be found online in the Supporting Information section at the end of this article.

## Figures and Tables

**FIGURE 1:**
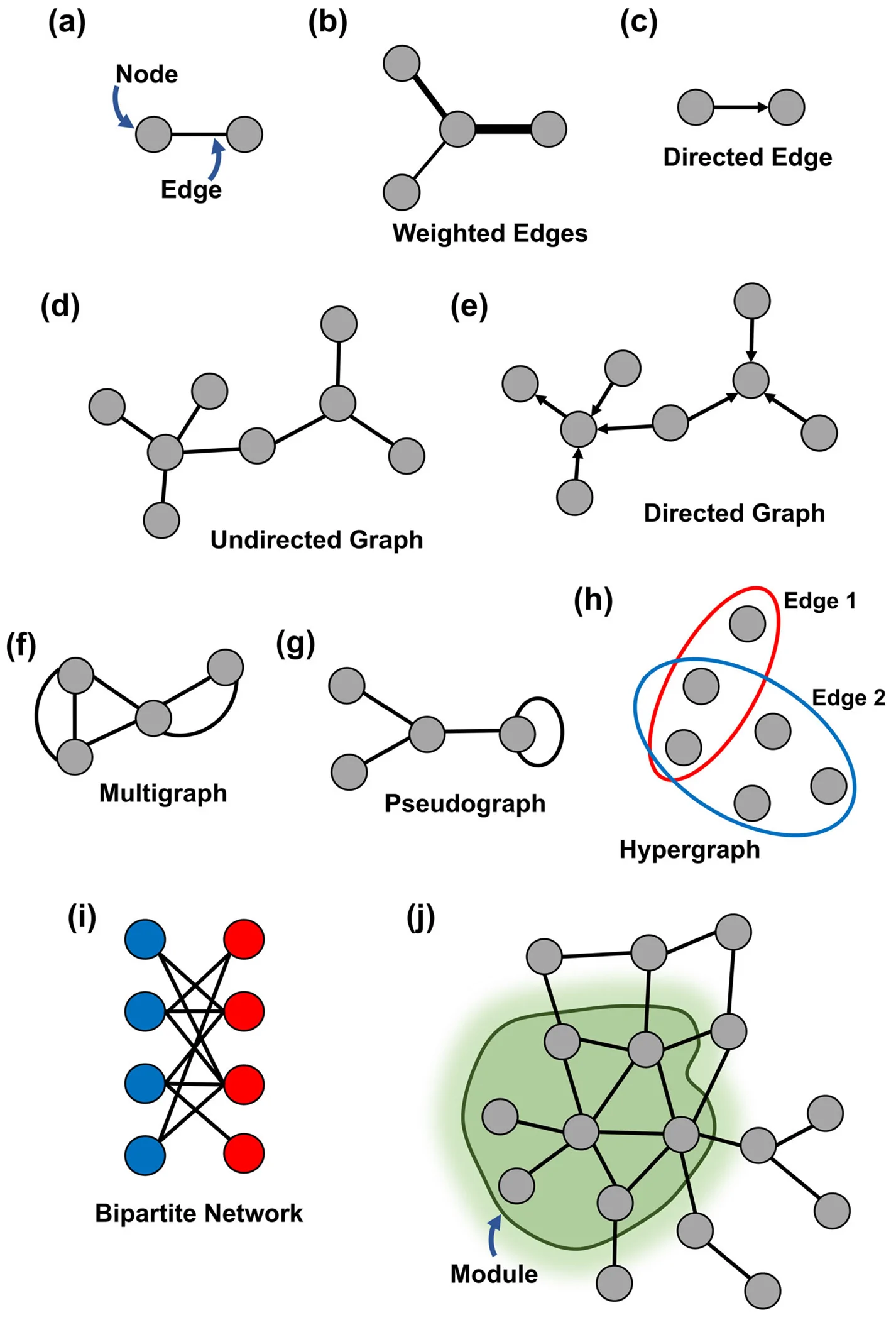
Basics of network representations. A network is composed of a group of nodes and their connections, (a) termed edges. Nodes and edges can represent any variety of information and the connections among them. (b) Edges can be weighted, indicating differences in strength or quality of the relationship between two nodes. (c) A directed edge conveys that information flows from one node to another in a particular direction. The representation of nodes and edges is termed a graph; these can be (d) undirected or (e) directed depending on whether directed edges are included in the graph. Variations in edge connections can result in several types of graphs. (f) A multigraph contains nodes connected by more than one edge. (g) A pseudograph contains nodes with an edge connecting back to the same node. (h) Hypergraphs have edges connecting more than two nodes. (i) A bipartite graph is composed of two groups of nodes (blue nodes and red nodes) with edges connecting nodes between the two groups but not within the same group. (j) Within a network space, functional and/or topological characteristics can be used to identify a subset of nodes which relate more closely to each other than to other nodes in the network. This subnetwork is called a module.

**FIGURE 2:**
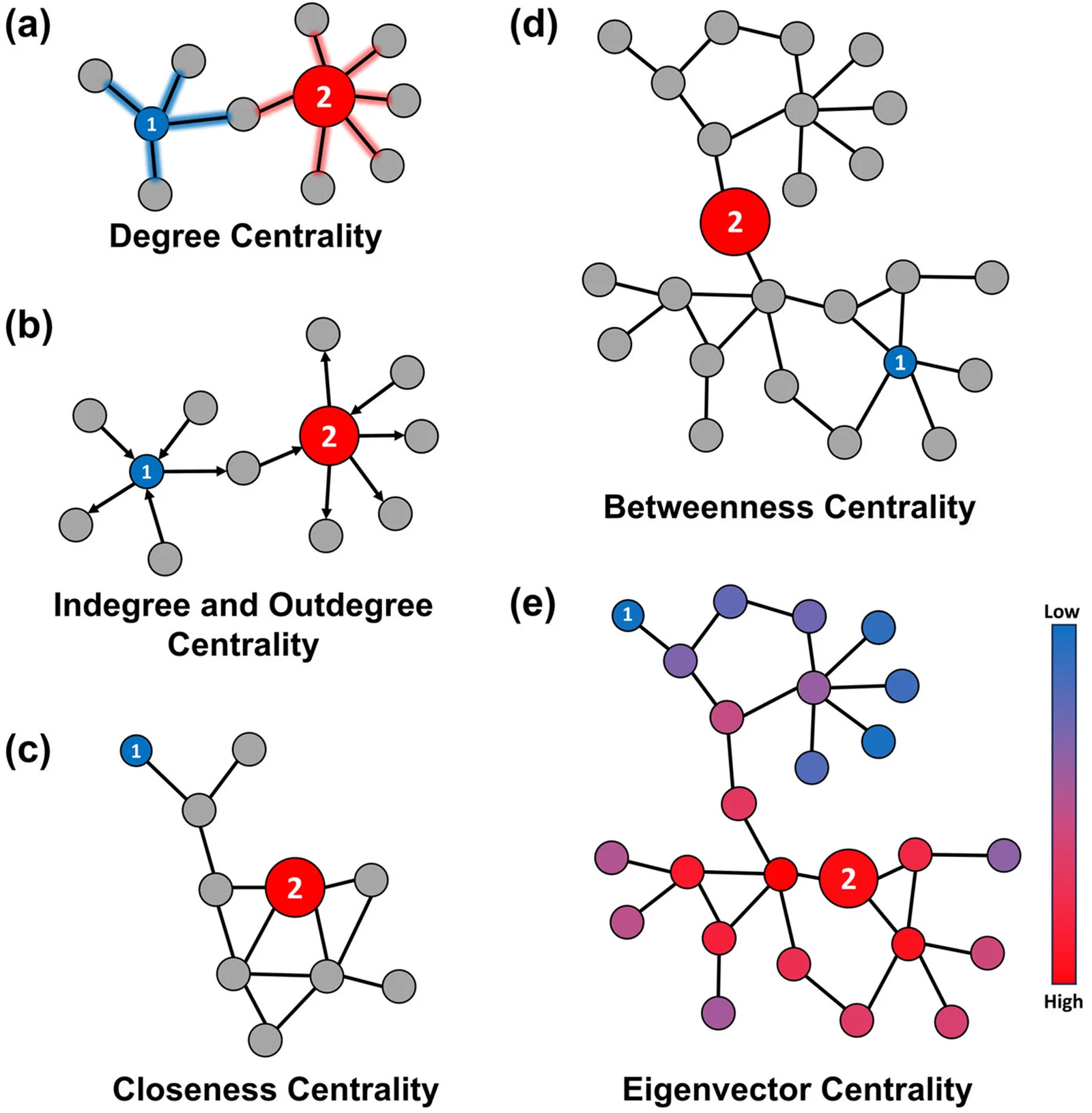
Measures of centrality. (a) Degree centrality is based on the degree, or number of connections (edges), of a node. Node 2 has a higher degree centrality (red edges) than node 1 (blue edges). (b) In directed networks, degree centrality can be further specified as indegree centrality, based on the number of incoming edges to a node, and outdegree, based on the number of outgoing edges. Node 2 has a higher outdegree centrality compared to node 1. (c) Closeness centrality is based on the shortest path distance from a node to all other nodes in the network. Node 2 has a higher closeness centrality than Node 1 because the path length in total to all nodes from Node 2 is smaller than from Node 1. (d) Betweenness centrality assesses what proportion of shortest paths between two nodes in a network include the node of interest. Node 2 has a greater betweenness centrality than Node 1 because a greater number of node pairs in the network would include Node 1 in the shortest path between the node pairs given its location at a critical junction in the network. (e) Eigenvector centrality identifies nodes that are connected to other nodes which are themselves highly connected. Using this conceptual formulation, nodes can be pseudo-coloured according to their relative eigenvector centrality (low to high represented as colours going from blue to red); Node 2 has a higher eigenvector centrality than Node 1.

**FIGURE 3:**
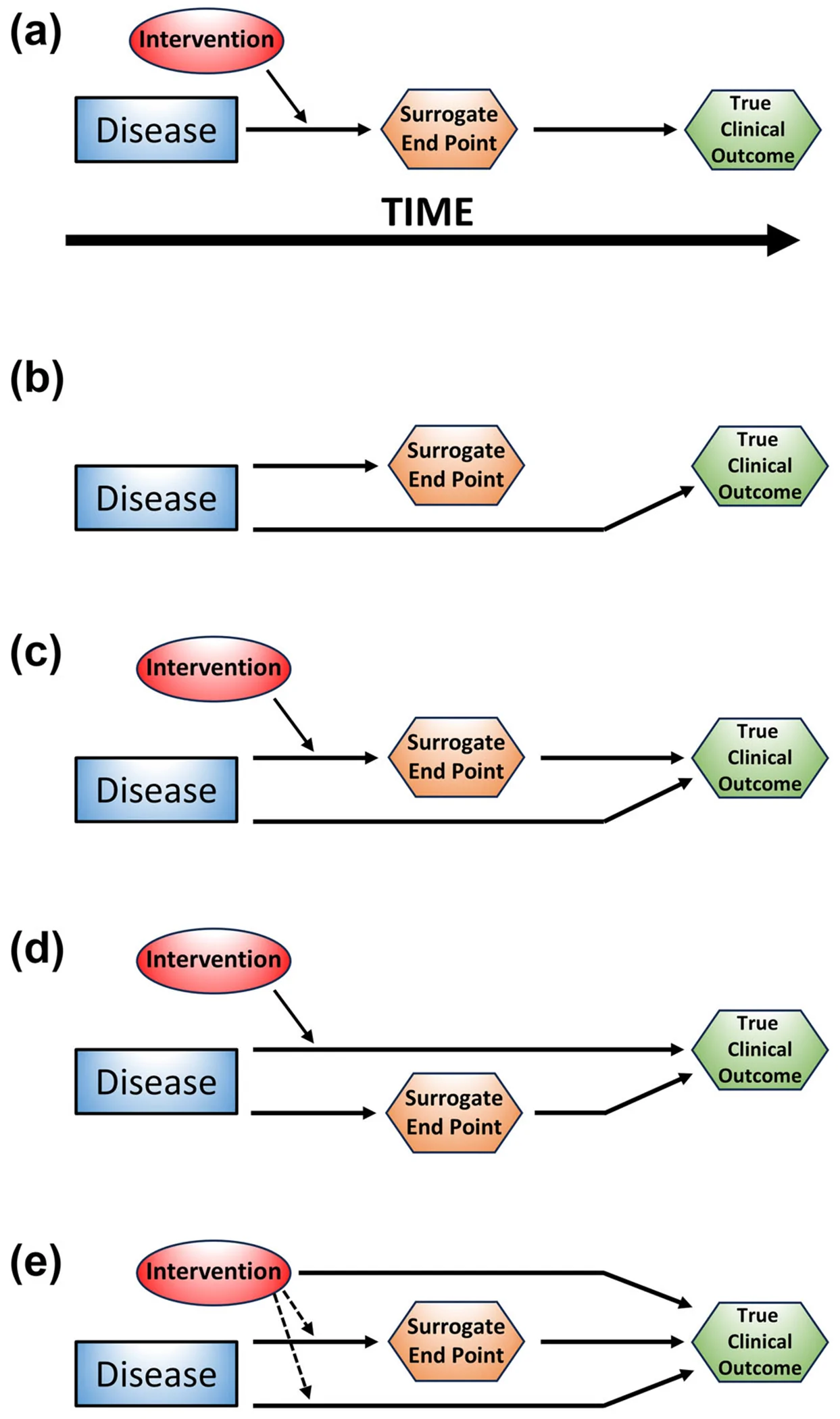
Biomarkers as surrogate endpoints. Biomarkers can be viewed as surrogate endpoints for a disease and disease-directed interventions, tracking with the clinical outcome of interest. However, (a) the idealised scenario where the biomarker (surrogate endpoint) is downstream in a linear fashion from the disease and intervention may not exist. Biological systems are often more complicated and can be represented by a range of relationships among the disease, intervention and biomarker. (b) The biomarker may not be in the causal pathway linking the disease and the true clinical outcome. The intervention of interest may only affect one pathway while a disease may have multiple pathways by which it leads to the clinical outcome. In such a scenario, a biomarker (c) may or (d) may not be in the pathway downstream of the intervention, making the biomarker a poor estimate for the net effect on the true clinical outcome. Alternatively, all of these scenarios may occur simultaneously, where the disease and the intervention both affect the outcome through mechanisms independent of the biomarker. Adapted from Fleming and DeMets (1996).

**FIGURE 4:**
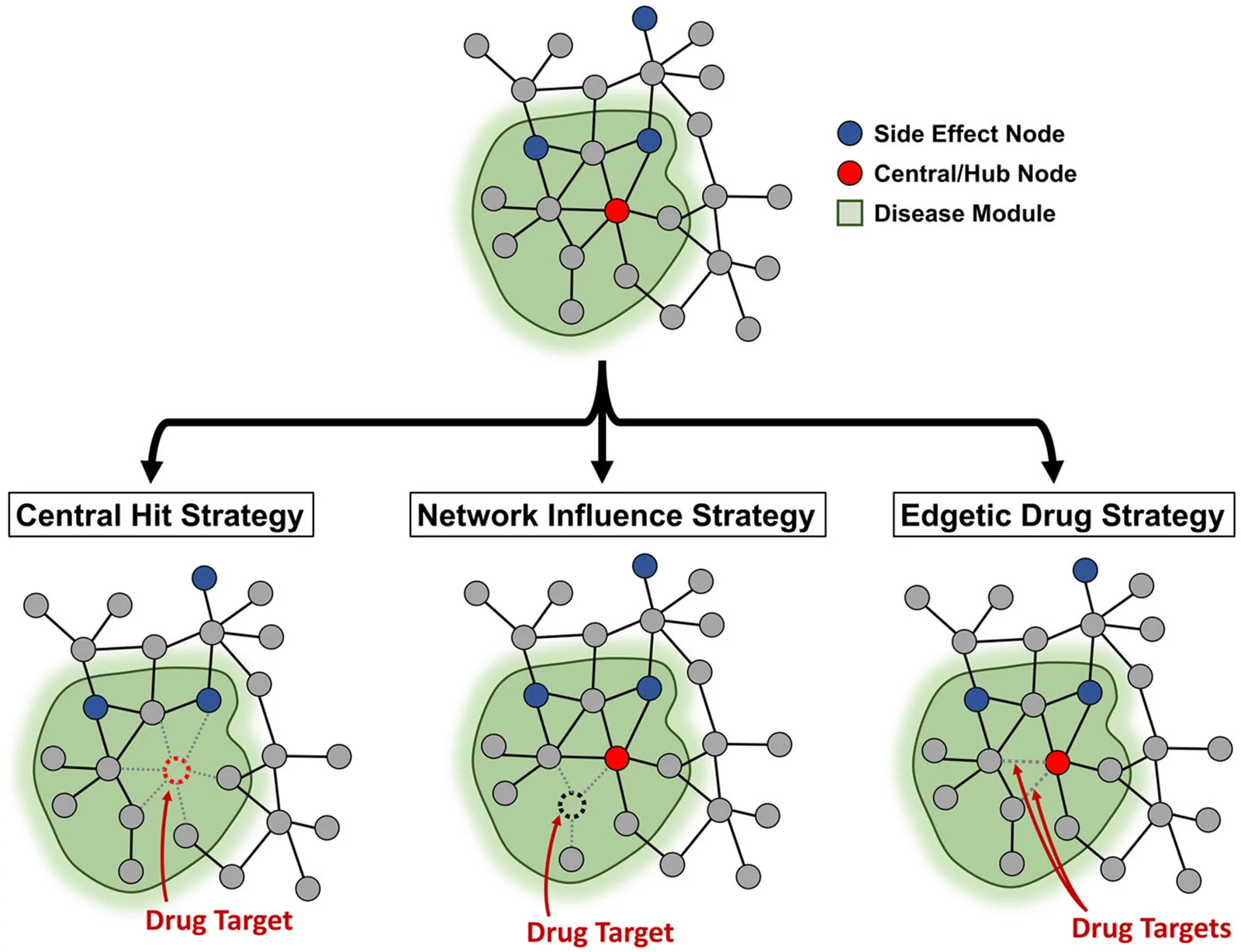
Network-informed drug targeting strategies. Different strategies for identifying drug targets can be selected based on the pathobiological scenario. A Central Hit Strategy targets a drug to a central node (hub) of a disease module, resulting in significant disruption of the underlying disease network structure and potentially affecting nodes associated with side effects. This type of strategy is useful for disease states characterised by high levels of robustness, flexibility and resiliency, such as cancer or infectious diseases. A Network Influence Strategy targets a drug to nodes that modify or influence a central node but do not target the high centrality node itself. This can decrease chances of impacting nodes associated with side effects. This strategy is useful for relatively rigid networks in which targeting a central node could result in significant disruption of the overall network architecture, resulting significant side effects or lethal toxicities in a biological system. An Edgetic Drug Strategy targets a drug to specific edges of a node, affecting a subset of a node’s interactions rather than the node in total. Such an approach could spare non-disease interactions of a node to limit side effects. In the context of protein–protein interaction networks, an edgetic drug would affect target protein binding with other protein partners.

**FIGURE 5:**
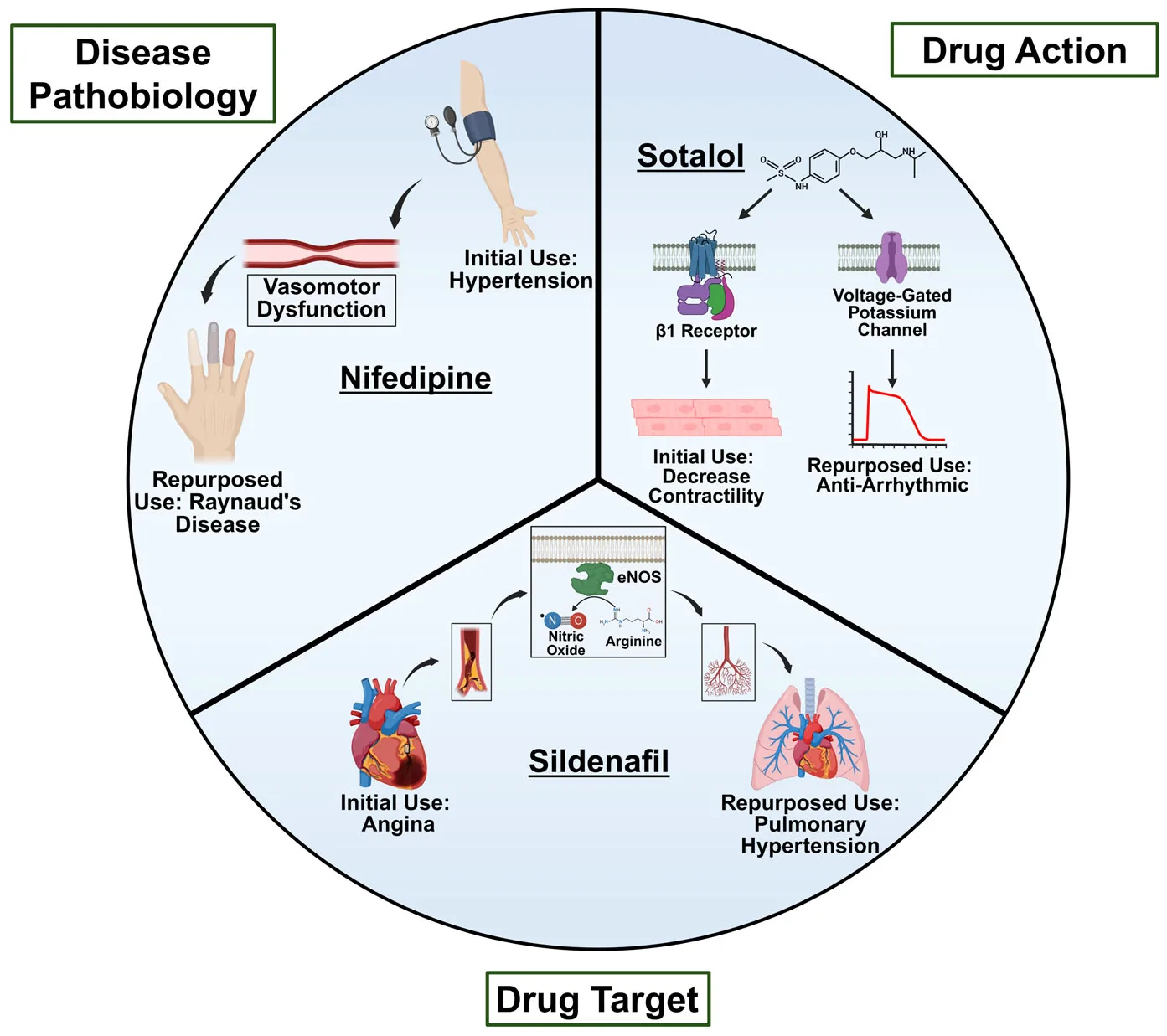
Drug repurposing strategies. Drug repurposing can be accomplished through several different strategies. A disease pathobiology approach uses common disease motifs to identify repositioning opportunities. Nifedipine, a calcium-channel blocker which counteracts vasomotor dysfunction to promote vasodilation in systemic hypertension, was repurposed for other diseases characterised by localised vasomotor dysfunction, such as Raynaud’s disease. A Drug Target strategy identifies a drug target which is common to another disease process; the drug for that common target can then be repurposed for the other disease. An example of this is sildenafil, which was initially studied for angina pectoris but which was subsequently repurposed for use in pulmonary hypertension. A Drug Action approach identifies new pharmacodynamic mechanisms by which a drug acts, allowing the drug to be used for new disease indications. This strategy was the basis for adapting sotalol, initially developed as a β_1_-adrenoceptor blocker to decrease myocyte contractility, for use as an anti-arrhythmic based on its additional pharmacodynamic effect of inhibiting voltage-gated potassium channels. Created in BioRender: https://BioRender.com, https://BioRender.com/t32n507.

## Data Availability

N/A as this is a review.

## Abbreviations:

AI: artificial intelligence
CMap: Connectivity Map
COVID-19: coronavirus disease 2019
GBA: guilt by association
miRNA: microRNA
MDM2: MDM2 proto-oncogene
PDE5: phosphodiesterase 5
RNA: ribonucleic acid
