# Supplementary material for: Network-based precision medicine and systems pharmacology
Source: Br J Pharmacol. Author manuscript; Available in PMC 2026 Nov 11. (PMC13754039; doi:10.1111/bph.70184)
Supplement: Supplementary file 1 [file NIHMS2219774-supplement-Pandey___Loscalzo_2026_Supplement.pdf]

Supplementary Table 1: Selected Methodologies Employed for Biomarker and Drug Discovery/Repurposing

Methodology	Notes	Year of Development	Software Availability?
**Biomarker Discovery**			
SPINNAKER[1]	Competing endogenous RNA (ceRNA) interactions	2022	Yes, open-source R package
Weighted gene coexpression network analysis (WGCNA)[2]	Module and hub gene identification	2008	Yes, open-source R package
Cytoscape with plugins including molecular complex detection (MCODE), clusterMaker, and cytoHubba[3-7]	Visualization and analyses of networks, including topological-based hub and module identification	2003	Yes, open-source
Pajek[8]	Visualization and analyses of networks, including centrality calculation for hub identification	1996	Yes, open-source
MicroRNA Enrichment Turned Network (MIENTURNET)[9]	Visualization and analysis of microRNA-target networks for biomarker identification	2019	Yes, online portal
Network-Based Stratification (NBS)[10,11]	Clusters tumor somatic mutations into network groupings that allow for clinical subtyping of patients	2013, repackaged in Python in 2018 (pyNBS)	Yes, open-source Python package
**Drug Target Identification**			
Network oriented Gene Entropy Approach (NOGEA)[12]	Identifies genes that have high likelihood to perturb disease networks	2021	Yes, open-source
Turbine[13]	Performs perturbation analysis and simulates network dynamics to identify drug targets that demonstrate a network influence strategy	2013	Unavailable
Cytoscape with plugin DyNet[3,14]	Visualization and analyses of networks, including calculation of rewiring metric based on gained or lost edges to identify edgetic drug targets	2003	Yes, open-source
**Drug Repurposing**			

SAveRUNNER[15,16]	Screens for novel potential indications of current drugs against diseases of interest by predicting new drug-disease associations based on network similarity; also attempts to maximize network distance between candidate drug targets and known side-effect genes	2021	Yes, open-source
SITAR[17]	Identifies rules that determine known drug-target interactions via logistic regression to then identify novel drug-target pairs	2011	No publicly available programmed implementation
PREDICT[18]	Identifies rules that determine known drug-disease interactions via logistic regression to then identify novel drug-disease pairs	2011	No publicly available programmed implementation
Connectivity Map (CMap)[19,20]	Compilation of gene expression profiles in response to drug- and gene-based perturbations	2006	Yes, online portal
DTINet[21]	Heterogenous network approach for predicting novel drug target interactions and drug repurposing candidates	2017	Yes, open source
Network-Based Drug Repurposing and Exploration (NeDRex)[22]	Platform for disease module discovery and drug repurposing using heterogenous networks created from several open-source biomedical databases and network-based algorithms including BiCoN[23] and DIAMOnD[24]	2021	Yes, open-source with online portal and Cytoscape app

Supplementary References

1. Paci P, Fiscon G. SPINNAKER: an R-based tool to highlight key RNA interactions in complex biological networks. *BMC Bioinformatics*. 2022;23:166. doi: 10.1186/s12859-022-04695-x
2. Langfelder P, Horvath S. WGCNA: an R package for weighted correlation network analysis. *BMC Bioinformatics*. 2008;9:559. doi: 10.1186/1471-2105-9-559
3. Shannon P, Markiel A, Ozier O, Baliga NS, Wang JT, Ramage D, Amin N, Schwikowski B, Ideker T. Cytoscape: a software environment for integrated models of biomolecular interaction networks. *Genome Res*. 2003;13:2498-2504. doi: 10.1101/gr.1239303
4. Morris JH, Apeltsin L, Newman AM, Baumbach J, Wittkop T, Su G, Bader GD, Ferrin TE. clusterMaker: a multi-algorithm clustering plugin for Cytoscape. *BMC Bioinformatics*. 2011;12:436. doi: 10.1186/1471-2105-12-436
5. Utriainen M, Morris JH. clusterMaker2: a major update to clusterMaker, a multi-algorithm clustering app for Cytoscape. *BMC Bioinformatics*. 2023;24:134. doi: 10.1186/s12859-023-05225-z
6. Bader GD, Hogue CW. An automated method for finding molecular complexes in large protein interaction networks. *BMC Bioinformatics*. 2003;4:2. doi: 10.1186/1471-2105-4-2
7. Chin CH, Chen SH, Wu HH, Ho CW, Ko MT, Lin CY. cytoHubba: identifying hub objects and sub-networks from complex interactome. *BMC Syst Biol*. 2014;8 Suppl 4:S11. doi: 10.1186/1752-0509-8-S4-S11
8. Mrvar A, Batagelj V. Analysis and visualization of large networks with program package Pajek. *Complex Adaptive Systems Modeling*. 2016;4:6. doi: 10.1186/s40294-016-0017-8
9. Licursi V, Conte F, Fiscon G, Paci P. MIENTURNET: an interactive web tool for microRNA-target enrichment and network-based analysis. *BMC Bioinformatics*. 2019;20:545. doi: 10.1186/s12859-019-3105-x
10. Hofree M, Shen JP, Carter H, Gross A, Ideker T. Network-based stratification of tumor mutations. *Nat Methods*. 2013;10:1108-1115. doi: 10.1038/nmeth.2651
11. Huang JK, Jia T, Carlin DE, Ideker T. pyNBS: a Python implementation for network-based stratification of tumor mutations. *Bioinformatics*. 2018;34:2859-2861. doi: 10.1093/bioinformatics/bty186
12. Guo Z, Fu Y, Huang C, Zheng C, Wu Z, Chen X, Gao S, Ma Y, Shahen M, Li Y, et al. NOGEA: A Network-oriented Gene Entropy Approach for Dissecting Disease Comorbidity and Drug Repositioning. *Genomics Proteomics Bioinformatics*. 2021;19:549-564. doi: 10.1016/j.gpb.2020.06.023
13. Szalay KZ, Csermely P. Perturbation centrality and turbine: a novel centrality measure obtained using a versatile network dynamics tool. *PLoS One*. 2013;8:e78059. doi: 10.1371/journal.pone.0078059
14. Goenawan IH, Bryan K, Lynn DJ. DyNet: visualization and analysis of dynamic molecular interaction networks. *Bioinformatics*. 2016;32:2713-2715. doi: 10.1093/bioinformatics/btw187
15. Fiscon G, Paci P. SAveRUNNER: an R-based tool for drug repurposing. *BMC Bioinformatics*. 2021;22:150. doi: 10.1186/s12859-021-04076-w
16. Paci P, Fiscon G, Conte F, Wang RS, Handy DE, Farina L, Loscalzo J. Comprehensive network medicine-based drug repositioning via integration of therapeutic efficacy and side effects. *NPJ Syst Biol Appl*. 2022;8:12. doi: 10.1038/s41540-022-00221-0
17. Perlman L, Gottlieb A, Atias N, Ruppin E, Sharan R. Combining drug and gene similarity measures for drug-target elucidation. *J Comput Biol*. 2011;18:133-145. doi: 10.1089/cmb.2010.0213

18. Gottlieb A, Stein GY, Ruppin E, Sharan R. PREDICT: a method for inferring novel drug indications with application to personalized medicine. *Mol Syst Biol*. 2011;7:496. doi: 10.1038/msb.2011.26
19. Lamb J, Crawford ED, Peck D, Modell JW, Blat IC, Wrobel MJ, Lerner J, Brunet JP, Subramanian A, Ross KN, et al. The Connectivity Map: using gene-expression signatures to connect small molecules, genes, and disease. *Science*. 2006;313:1929-1935. doi: 10.1126/science.1132939
20. Subramanian A, Narayan R, Corsello SM, Peck DD, Natoli TE, Lu X, Gould J, Davis JF, Tubelli AA, Asiedu JK, et al. A Next Generation Connectivity Map: L1000 Platform and the First 1,000,000 Profiles. *Cell*. 2017;171:1437-1452 e1417. doi: 10.1016/j.cell.2017.10.049
21. Luo Y, Zhao X, Zhou J, Yang J, Zhang Y, Kuang W, Peng J, Chen L, Zeng J. A network integration approach for drug-target interaction prediction and computational drug repositioning from heterogeneous information. *Nat Commun*. 2017;8:573. doi: 10.1038/s41467-017-00680-8
22. Sadegh S, Skelton J, Anastasi E, Bernett J, Blumenthal DB, Galindez G, Salgado-Albarran M, Lazareva O, Flanagan K, Cockell S, et al. Network medicine for disease module identification and drug repurposing with the NeDRex platform. *Nat Commun*. 2021;12:6848. doi: 10.1038/s41467-021-27138-2
23. Lazareva O, Canzar S, Yuan K, Baumbach J, Blumenthal DB, Tieri P, Kacprowski T, List M. BiCoN: network-constrained biclustering of patients and omics data. *Bioinformatics*. 2021;37:2398-2404. doi: 10.1093/bioinformatics/btaa1076
24. Ghiassian SD, Menche J, Barabasi AL. A DIseAse MOdule Detection (DIAMOnD) algorithm derived from a systematic analysis of connectivity patterns of disease proteins in the human interactome. *PLoS Comput Biol*. 2015;11:e1004120. doi: 10.1371/journal.pcbi.1004120
